# A negative binomial regression model for risk estimation of 0–2 axillary lymph node metastases in breast cancer patients

**DOI:** 10.1038/s41598-020-79016-4

**Published:** 2020-12-14

**Authors:** De Zeng, Hao-Yu Lin, Yu-Ling Zhang, Jun-Dong Wu, Kun Lin, Ya Xu, Chun-Fa Chen

**Affiliations:** 1 Department of Medical Oncology, Cancer Hospital of Shantou University Medical College, Shantou, 515031 China; 2 Guangdong Provincial Key Laboratory of Breast Cancer Diagnosis and Treatment, Shantou, 515031 China; 3 Department of Thyroid and Breast Surgery, The First Affiliated Hospital of Shantou University Medical College, Shantou, 515041 China; 4 Department of Information, Cancer Hospital of Shantou University Medical College, Shantou, 515031 China; 5 The Breast Center, Cancer Hospital of Shantou University Medical College, Shantou, 515031 China; 6 Department of Public Health and Preventive Medicine, Shantou University Medical College, Shantou, 515041 China

**Keywords:** Breast cancer, Cancer models

## Abstract

Extensive clinical trials indicate that patients with negative sentinel lymph node biopsy do not need axillary lymph node dissection (ALND). However, the ACOSOG Z0011 trial indicates that patients with clinically negative axillary lymph nodes (ALNs) and 1–2 positive sentinel lymph nodes having breast conserving surgery with whole breast radiotherapy do not benefit from ALND. The aim of this study is therefore to identify those patients with 0–2 positive nodes who might avoid ALND. A total of 486 patients were eligible for the study with 212 patients in the modeling group and 274 patients in the validation group, respectively. Clinical lymph node status, histologic grade, estrogen receptor status, and human epidermal growth factor receptor 2 status were found to be significantly associated with ALN metastasis. A negative binomial regression (NBR) model was developed to predict the probability of having 0–2 ALN metastases with the area under the curve of 0.881 (95% confidence interval 0.829–0.921, *P* < 0.001) in the modeling group and 0.758 (95% confidence interval 0.702–0.807, *P* < 0.001) in the validation group. Decision curve analysis demonstrated that the model was clinically useful. The NBR model demonstrated adequate discriminative ability and clinical utility for predicting 0–2 ALN metastases.

## Introduction

The number of axillary lymph node (ALN) metastases is an essential indicator for evaluating the severity and survival of patients with breast cancer^1^. A greater number of positive ALNs is associated with higher probability of recurrence and mortality^2^, and more than 6 metastatic ALNs might indicate a high risk of distant metastasis^3^. The number of involved ALNs is a determinant parameter in treatment decision making, particularly for those with 1–2 positive sentinel lymph nodes (SLNs). For these subgroups of patients and those with negative ALNs, sentinel lymph node biopsy (SLNB) has now become the preferred surgical procedure, rather than axillary lymph node dissection (ALND)^4–6^.

A number of studies have shown that, in the absence of preoperative ALN status evaluation, including those with T1–4 stage diseases, approximately 51–67% of patients with breast cancer have negative ALNs^7–10^. About 40% of patients with clinically suspicious ALN metastasis had pathologically negative SLNs^11–13^. For almost half of the patients with involved SLNs, there was no addition of involved ALNs identified on ALND^14,15^. Even in the T1–2 stage patients with positive SLNs, approximately 60–73% of patients had no additional non-SLN metastasis after ALND^4,6^. Extensive clinical trials indicate that patients with negative SLNB do not need ALND, and the ACOSOG Z0011 trial showed that patients with clinical T1–2N0 tumors, without palpable lymph adenopathy, and 1–2 positive SLNs receiving breast conserving surgery and subsequent radiation and adjuvant therapy could be spared from ALND^6,16^. Therefore, the development of reliable predictors to identify those patients with 0–2 positive nodes who might avoid ALND is needed for treatment quality assurance and improvement.

Several studies have used preoperative ultrasound imaging^17^, computed tomography imaging^18^, magnetic resonance imaging^17^, or ultrasound-guided fine-needle biopsy to evaluate the status of ALNs^19^. However, these studies often neglected the distribution of involved nodes and usually investigated the axillary status using a logistic regression model for predicting ALN status, which is not conducive to predicting the number of metastatic nodes. The numbers of metastatic ALNs are count data that often display overdispersion^20^; hence, negative binomial regression (NBR), which has been well described by Hilbe^21^, is an appropriate choice for modeling this distribution^20,22,23^. A recent study comprising of 109,618 patients with breast cancer from the Surveillance, Epidemiology, and End Results database indicated that the distribution of overdispersion of metastatic ALNs was similar to a log-concave curve^23^. The NBR model provided an excellent fit curve, which could predict the number of involved nodes. In this retrospective study, we aim to first identify factors that affect counts of ALN metastasis and then develop an NBR model to estimate the probability of exact counts of ALN metastasis and calculate the probability of 0–2 ALN metastases, with the ultimate goal of guiding physicians to make appropriate surgical plans preoperatively for breast cancer patients.

## Results

### Patients characteristic

The demographic information and descriptive statistics are summarized in Table 1. The estrogen receptor (ER) status and Ki67 index between the modeling and the validation groups was significantly different (*P* < 0.05). There were more patients with ER-negative and Ki67 ≥ 14% in the modeling group than in the validation group. A significant difference was also found in the type of axillary surgery between these two groups (*P* < 0.001), with more patients receiving SLNB in the validation group than in the modeling group. Considering the marked difference in the counts of metastatic nodes in patients who underwent SLNB + ALND between the two groups (*P* < 0.05), more metastatic nodes were dissected in the validation group than in the modeling group. The counts of dissected nodes between the two groups were significantly different (*P* < 0.05), and more nodes were dissected in the modeling group than in the validation group. The counts associated with SLNB were also different between the modeling group and the validation group (*P* < 0.05), and more SLNs were dissected in the validation group than in the modeling group. No significant difference was found between the modeling group and the validation group in terms of the other clinicopathologic variables (*P* > 0.05).

**Table 1:** Comparison of descriptive characteristics between the modeling group and the validation group.

Characteristics	Modeling group		Validation group		*P*
	No	%	No	%	
No. of patients	212	100.0	274	100.0	
Female	212	100.0	274	100.0	
Age (years)					
Median (range)	50.5 (23–91)		50 (24–84)		0.916
Menstrual status					0.373
Pre-menopause	109	51.4	152	55.5	
Post-menopause	103	48.6	122	44.5	
Laterality					0.414
Left	117	55.2	141	51.5	
Right	95	44.8	133	48.5	
Tumor size (cm)					
Median (range)	3.0 (0.7–9.0)		3.0 (1.0–10.0)		0.756
Tumor location					0.143
UOQ	99	46.7	156	56.9	
LOQ	21	9.9	27	9.9	
LIQ	21	9.9	15	5.5	
UIQ	37	17.5	40	14.6	
CR	34	16.0	36	13.1	
Clinical ALN status					0.056
Negative	103	48.6	157	57.3	
Positive	109	51.4	117	42.7	
Stage					0.083
I	36	17.0	59	21.5	
II	116	54.7	160	58.4	
III	60	28.3	55	20.1	
Histologic grade					0.161
1–2	135	63.7	191	69.7	
3	77	36.3	83	30.3	
ER status					0.036
Negative	81	38.2	80	29.2	
Positive	131	61.8	194	70.8	
PR status					0.435
Negative	91	42.9	108	39.4	
Positive	121	57.1	166	60.6	
HER2 status					0.707
Negative	145	68.4	183	66.8	
Positive	67	31.6	91	33.2	
Ki67					0.048
< 14%	21	9.9	44	16.1	
≥ 14%	191	90.1	230	83.9	
Molecular subtype					0.136
HR(+)/HER2(−)	109	51.4	142	51.8	
HR(+)/HER2(+)	29	13.7	56	20.4	
TN	36	17.0	41	15.0	
HER2(+)	38	17.9	35	12.8	
Breast surgery					0.463
Conserving surgery	50	23.6	57	20.8	
Mastectomy	162	76.4	217	79.2	
Axillary surgery					< 0.001
SLNB	56	26.4	121	44.2	
SLNB + ALND	43	20.3	66	24.1	
ALND	113	53.3	87	31.7	
ALNs metastasis					0.248
Negative	102	48.1	148	54.0	
1–2 positive ALNs	44	20.8	59	21.5	
> 2 positive ALNs	66	31.1	67	24.5	
Counts of metastatic nodes (median, rang)	1 (0–35)		0 (0–29)		0.125
SLNB	0 (0–1)		0 (0–2)		0.824
SLNB + ALND	1 (0–10)		2 (0–29)		0.002
ALND	3 (0–35)		1 (0–26)		0.537
Counts of dissected nodes (median, rang)	15 (1–43)		9 (1–32)		0.004
SLNB	2 (1–5)		2 (1–7)		0.036
SLNB + ALND	17.5 (8–32)		17 (6–30)		0.461
ALND	17 (2–43)		18 (7–32)		0.584

Abbreviation: ALN, Axillary lymph node; ALND, axillary lymph node dissection; CR, Central region; ER, estrogen receptor; HER2, human epidermal growth factor receptor 2; HR, hormone receptor; LIQ, Lower inner quadrant; LOQ, Lower outer quadrant; PR, progesterone receptor; SLN, sentinel lymph node; SLNB, sentinel lymph node biopsy; TN, triple negative; UIQ, Upper inner quadrant; UOQ, Upper outer quadrant.

The distribution of ALNs in the modeling group and the validation group are shown in Table 2. There were 48.1% of patients with negative ALNs and 20.7% of patients with 1–2 ALNs metastases in the modeling group compared to 54.0% of patients with negative ALNs and 21.4% of patients with 1–2 ALNs metastases in the validation group.

**Table 2:** Distribution of metastatic axillary lymph nodes.

Count of metastatic axillary lymph nodes	Modeling group	Validation group
	No. (%) (n = 212)	No. (%) (n = 274)
0	102 (48.1)	148 (54.0)
1	27 (12.7)	40 (14.5)
2	17 (8.0)	19 (6.9)
3	15 (7.0)	19 (6.9)
4	12 (5.7)	9 (3.3)
5	5 (2.4)	3 (1.1)
6	8 (3.8)	6 (2.2)
7	4 (1.9)	4 (1.5)
8	7 (3.3)	4 (1.5)
9	1 (0.5)	4 (1.5)
10	3 (1.4)	–
11	2 (0.9)	2 (0.7)
12	3 (1.4)	3 (1.1)
13	2 (0.9)	2 (0.7)
18	–	1 (0.4)
19	–	1 (0.4)
20	–	2 (0.7)
21	–	2 (0.7)
22	–	1 (0.4)
24	1 (0.5)	–
26	1 (0.5)	2 (0.7)
27	–	1 (0.4)
28	1 (0.5)	–
29	–	1 (0.4)
35	1 (0.5)	–

### The goodness of fit for a negative binomial distribution and Poisson distribution

**Figure 1:**
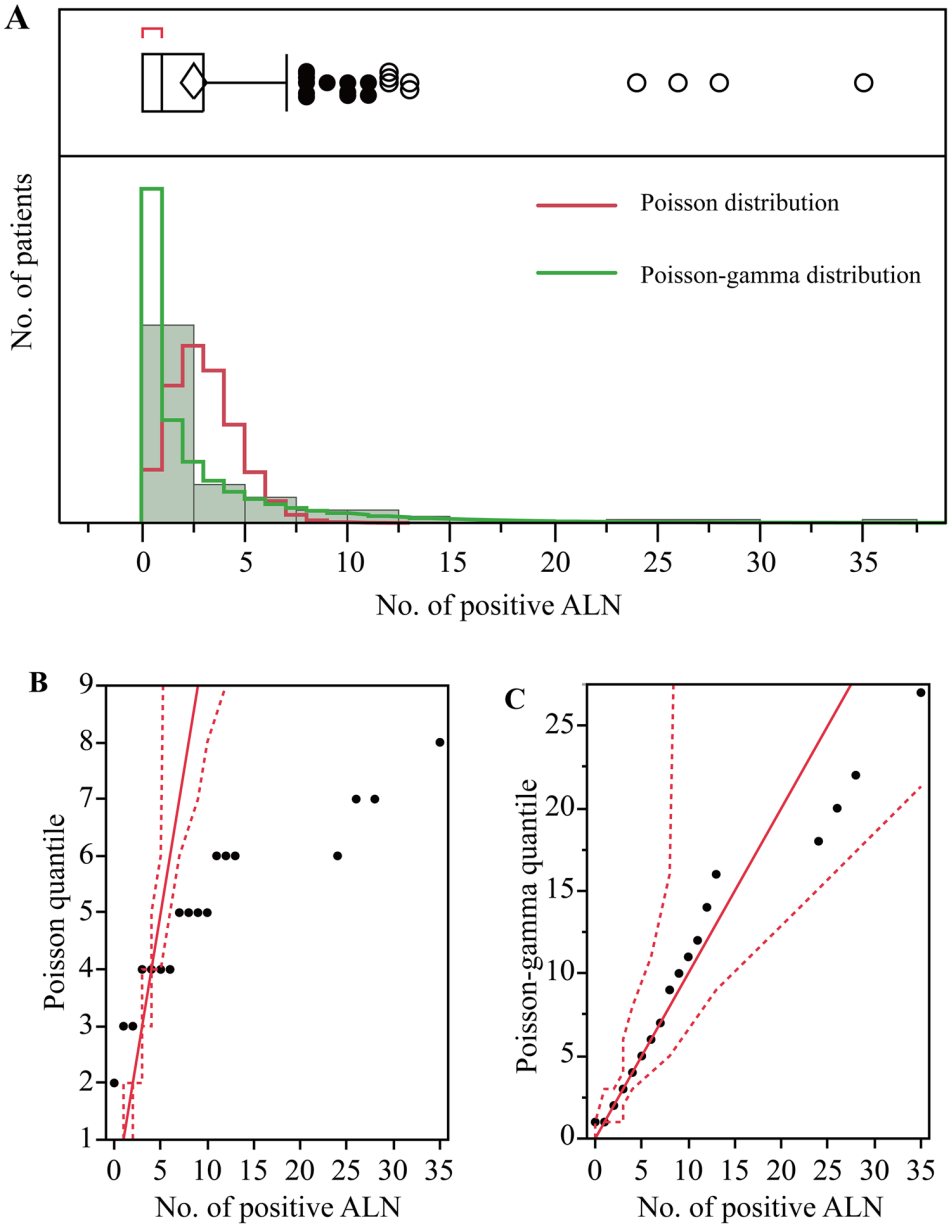
Distribution of metastatic axillary lymph node (ALN) counts. (**A**) Distribution of the metastatic ALNs. The counts of metastatic ALNs approximately follow a Poisson-gamma distribution. In the boxplot above the graph, the vertical line in the bottom of the box indicates the median, the diamond indicates the mean, and the left and right borders of the box mark the 25th and 75th percentiles, respectively. The solid point extending from the right of the box marks the end of 95th percentiles. The hollow points are outliers beyond the 95th percentile. (**B**) Goodness of fit with a Poisson distribution. The red line indicates the fit line and the dotted line shows the 95% confidence interval. (**C**) Goodness of fit with Poisson-gamma distribution. The red line indicates the fit line, and the dotted line shows the 95% confidence interval.

The histograms and fitted curves of the number of ALNs metastases in the present study are shown in Fig. 1A. The mean and median numbers of metastatic ALNs were 2.58 (variance of 22.38) and 1 (range 0 to 35), respectively, in modeling group. The distribution showed a logarithmic concave curve, suggesting that the majority of patients had negative ALNs and then decreased as the number of metastatic ALNs increased. It could be fitted with a Poisson distribution or a Poisson-gamma distribution. However, the goodness of fit with Poisson-gamma distribution was better than that with the Poisson distribution (Fig. 1B,C). In addition, the variance of the number of metastatic ALNs in the present study was nearly 10 times greater than the mean, and the NBR model provided an improved fit for the data and accounted better for overdispersion than did the Poisson regression model, which assumes that the mean and variance are the same. Therefore, we adopted the NBR model to analyze the data in the present study. The lack-of-fit statistics with the NBR model in the modeling group are shown in Table S1.

### The result of the negative binomial regression model

As shown in Table 3, several factors were independently correlated with the counts of metastatic ALNs, including clinical lymph node status, histologic grade, ER status, and human epidermal growth factor receptor 2 (HER2) status. The incidence rate ratio (IRR) of positive clinical lymph node status was higher for positive ALN metastasis [IRR = 2.88, 95% confidence interval (CI) 2.29–3.63] than for negative clinical lymph node status. A similar result was also found for histologic grade; the IRR of ALN metastasis in histologic grade 3 was 1.38 (95% CI 1.14–1.69) times higher than that in histologic grade 1–2 patients. Similarly, the IRR of ALN metastasis in HER2-positive patients was 1.33 (95% CI 1.06–1.67) times higher than that in HER2-negative patients. Patients who were ER-positive were more likely to suffer ALN metastasis than ER-negative patients, and the IRR of ALN metastasis in ER-positive patients was 1.48 (95% CI 1.18–1.87) times higher than thar in ER-negative patients. Although there was no statistical difference between the size of primary tumor and the number of ALNs involvement in the model, there was a trend for tumor size to be associated with the number of ALNs metastases. The larger the tumor was, the higher the risk of ALNs metastases. For every 1 cm increase in the diameter of the tumor, the risk increased by 0.13 times.

**Table 3:** The fitting result of negative binomial regression model.

Independent variable	Coefficients	Standard error	Wald χ2	*P*	IRR (95% CI)
Intercept	− 0.03	0.26	− 0.13	0.899	0.97 (0.58–1.60)
**Clinical lymph node status**					
Negative	–	–	–	–	–
Positive	1.06	0.12	9.00	< 0.001	2.88 (2.29–3.63)
**Histologic grade**					
1–2	–		–	–	–
3	0.33	0.10	3.22	0.001	1.38 (1.14–1.69)
**ER status**					
Negative	–	–	–	–	–
Positive	0.39	0.12	3.36	< 0.001	1.48 (1.18–1.87)
**HER2 status**					
Negative	–		–	–	–
Positive	0.28	0.12	2.42	0.016	1.33 (1.06–1.67)
Primary tumor size	0.12	0.07	1.78	0.075	1.13 (0.99–1.29)

Abbreviation: CI, confidence interval; ER, estrogen receptor; HER2, human epidermal growth factor receptor 2; IRR, Incidence rate ratio.

**Figure 2:**
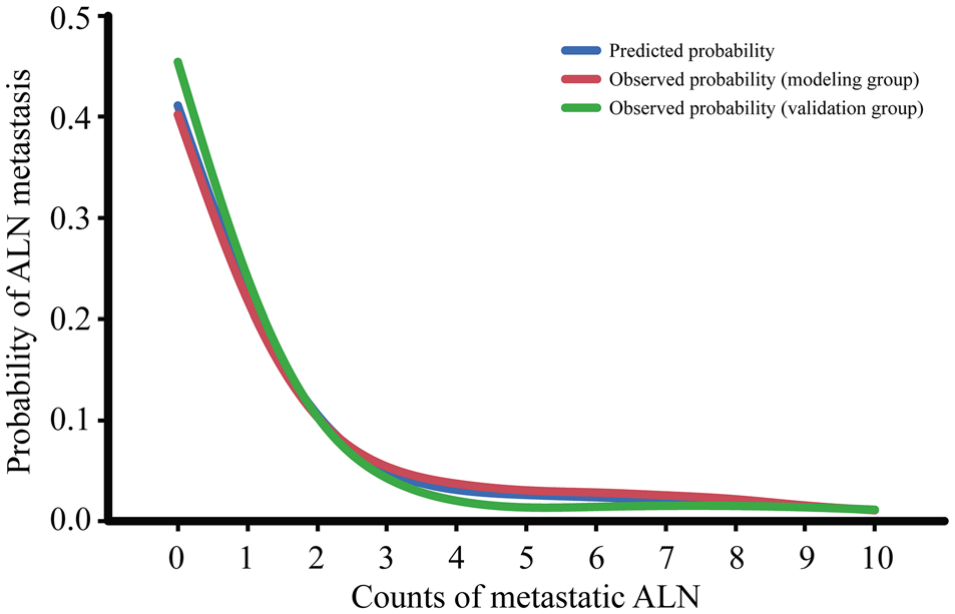
The probabilistic distribution of the negative binomial regression model fitted by the predicted group and observed group. ALN, axillary lymph node.

As shown in Fig. 2, the probabilistic distribution of the NBR model fit both the modeling group and the validation group well, and there were no statistically significant differences between the predicted and the observed probability. The prediction between the observed modeling group and the validation group were *P* = 1.0 and *P* = 0.736, respectively.

### The discrimination and clinical utility of the NBR model for predicting 0–2 ALN metastases

**Figure 3:**
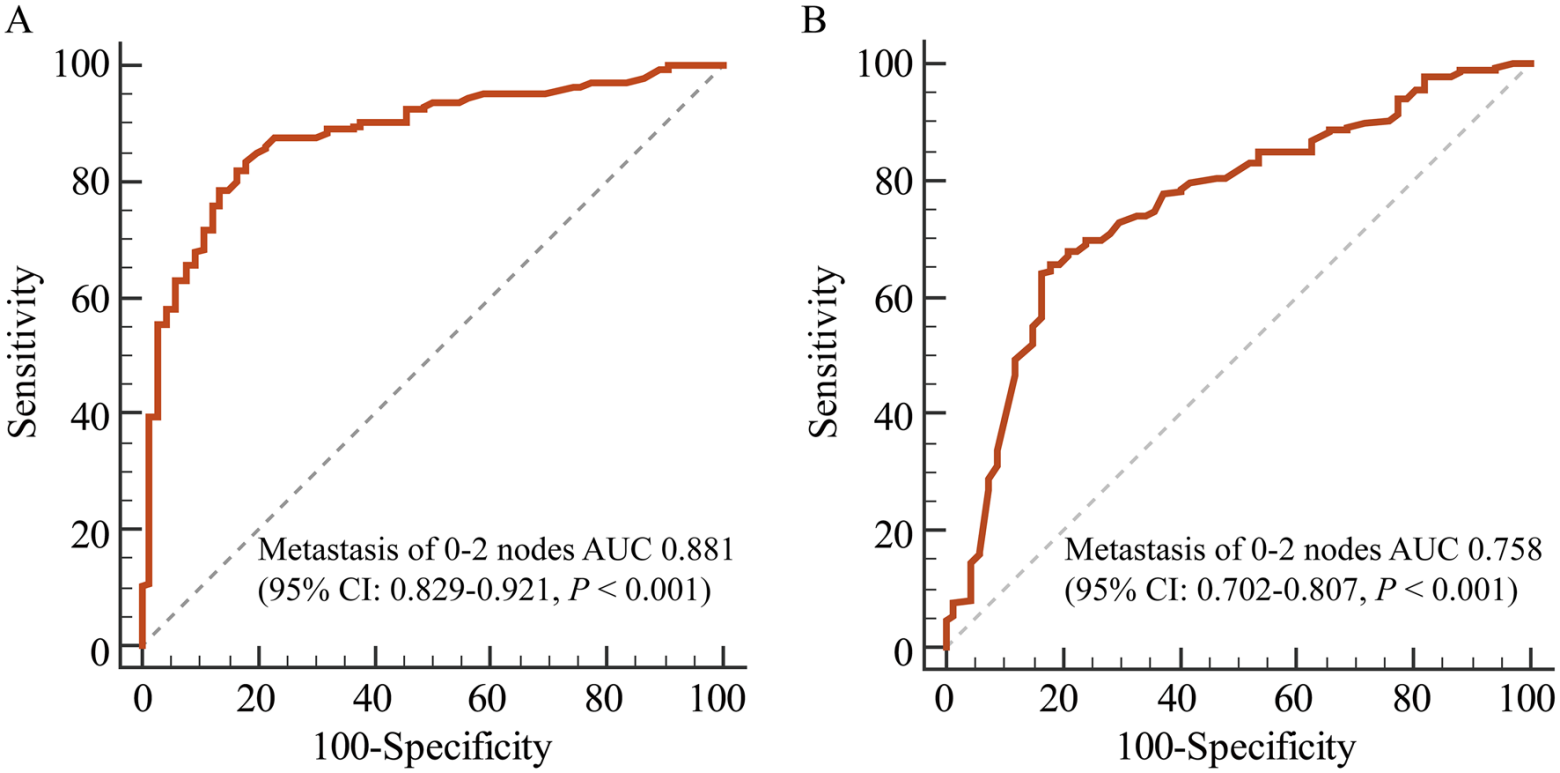
Receiver operating characteristic curve corresponding to the negative binomial regression model. (**A**) Area under the curve (AUC) for predicting 0–2 metastatic nodes in the modeling group. (**B**) AUC for predicting 0–2 metastatic nodes in the validation group. CI, confidence interval.

In the modeling group, the Area under the curve (AUC) for predicting 0–2 ALN metastases was 0.881 (95% CI 0.829–0.921; *P* < 0.001), and the corresponding sensitivity and specificity were 82.2% and 83.3%, respectively (Fig. 3A). In the validation group, the optimal cutoff value of probability was 90.0%, with an AUC for predicting 0–2 ALN metastases was 0.758 (95% CI 0.702–0.807; *P* < 0.001). The sensitivity, specificity and Yondex index were 64.3%, 83.6% and 47.8%, respectively (Fig. 3B). Both of the results indicated a good predictive ability of the NBR model.

**Figure 4:**
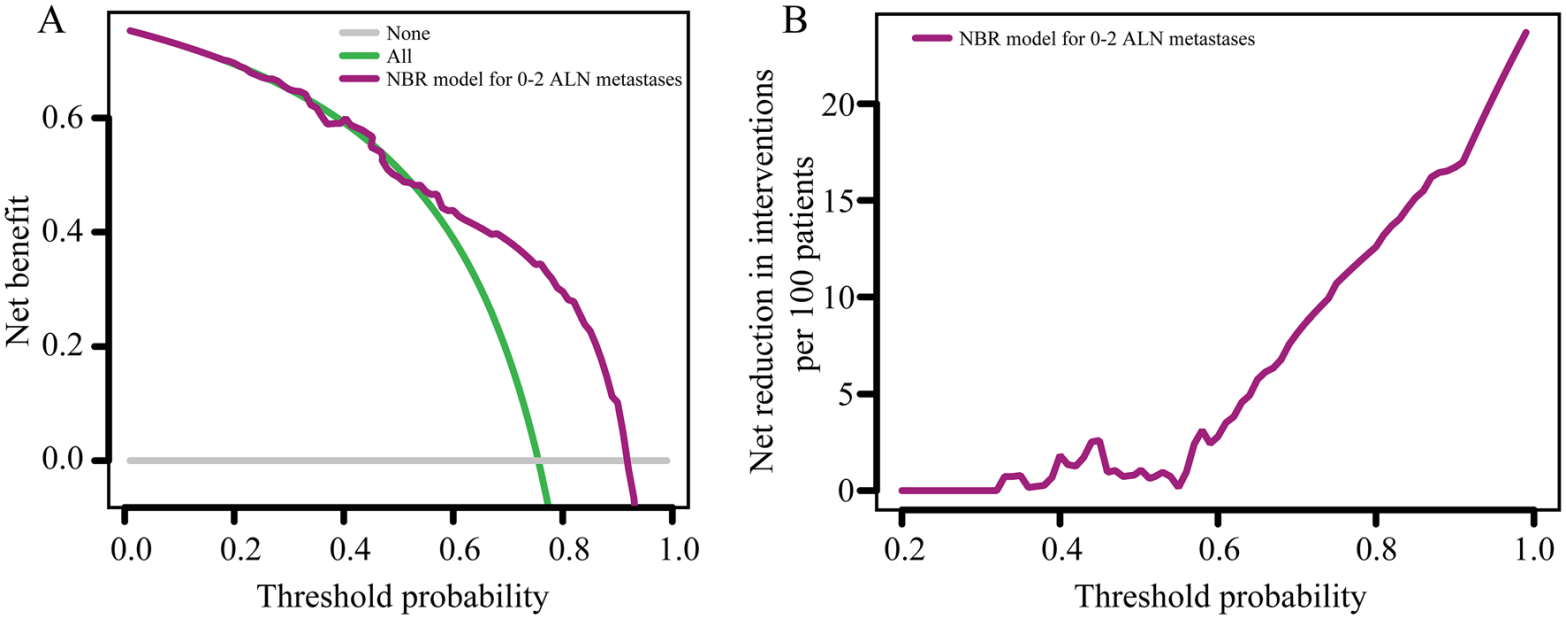
Decision curve analysis of the negative binomial regression (NBR) model in the validation group. (**A**) Net benefits for predicting 0–2 axillary lymph node (ALN) metastases. (**B**) Net reduction in interventions per 100 patients at different probability thresholds for predicting 0–2 ALN metastases.

The decision curve analysis (DCA) showed that the net benefit with the NBR model for predicting 0–2 ALN metastases in the validation group, with a probability range of 0.58 to 0.90, was superior to either the treat-all or treat-none methods (Fig. 4A). For example, the net benefit was approximately 11% at the 90% probability threshold, which indicated that at this probability threshold was equivalent to indicating 11 patients with 0–2 ALN metastases per 100 patients. At a probability threshold of 90%, the net reduction in interventions was approximately 16 per 100 patients (Fig. 4B). For example, this probability threshold is equivalent to 16 per 100 patients have 0–2 ALN metastases, which may indicate that 16% of patients can avoid unnecessary ALND.

## Discussion

Assessment of axillary status has become a fundamental step in cancer counseling and treatment planning in clinical practice, which might identify low- or high-risk situations and aid in treatment decision making, such as the choice between SLNB or neoadjuvant therapy^24^. At present, most of the available studies, which addressed the issue of prediction of ALN status, adopted the categorical (dichotomous/polychotomous) outcome variables and applied binary/multinomial logistic regression models to find their predictors^9,19^. These studies with logistic regression models could merely predict ALN status, such as negative or positive results, while neglecting the actual distribution of the involved nodes. Therefore, we argued that a method of modeling that takes into account the number of nodes, in combination with the nodal status, would allow for a grading of nodal involvement risk and might identify candidate patients for whom SLNB, neoadjuvant chemotherapy, ALND or radiotherapy be justified^23^. Especially after the ACOSOG Z0011 trial, the treatment strategy for axillary surgery according to the number of metastatic ALNs has become particularly important.

A large database composed of 224,656 breast cancer, 12,404 gastric cancer, 18,015 rectal cancer, 4,117 cervical cancer, 2,443 laryngeal cancer and 9,118 melanoma patients indicated that the number of involved lymph nodes is a set of nonnegative integer values not normally distributed, and the data exhibited considerable heterogeneity within populations^20^. Consistent with this phenomenon, the frequency distributions for the numbers of involved ALNs in the present study appeared skewed, with a large proportion of individuals with no involved nodes and a smaller proportion with many involved nodes. A statistical description of the frequency distribution of the numbers of involved nodes in an affected population could potentially reveal mechanisms of axillary metastasis, and eventually facilitate predictive models for axillary sampling^22^. Therefore, we adopted the NBR model for our study. In line with our study, Guern et al. indicated that the NBR model provided a better fit for the number of metastatic ALNs in breast cancer than the Poisson regression model^23^.

At present, the exact mechanism of ALN metastasis is unclear. In clinical practice, some patients are more susceptible to ALN metastasis than would be suggested by random events. Our results showed that the variance in the number of metastatic ALNs was nearly 10 times greater than the mean, indicating that the number of involved ALNs in patients with breast cancer is highly variable. This overdispersion of involved ALNs was described by Guern and colleagues and is known as "infectivity", that is, the more involved ALNs a person has, the greater the risk that the same person will subsequently have more involved ALNs^23^. Bori and colleagues also described this phenomenon in colorectal cancer^25^. A cascade process, which we believe is more appropriate than an infection-type process, is easy to understand because lymph nodes are not isolated organs but are interconnected through a complete lymphatic network. In addition, overdispersion means that, as the number of lymph nodes increases, the variance increases. In addition, when the risk increases, individual variability also increases. There are still some clinical paradoxes in that some patients with severe ALN metastasis do not end up with distant metastasis and have a relatively favorable prognosis.

Primary tumor size is the most powerful predictor of ALN status. There is a linear relationship between tumor size and ALN involvement. The chance of ALN involvement significantly increased from pT1mic (9.5%) to pT2 and pT3 tumor specimens (49.9% and 65.6%, respectively)^10^. Although tumor size was not significantly associated with the counts of ALN metastases in our study, it could not be excluded in our model. Our results showed a trend that the risk of ALN metastases increases consistently with tumor size increases. This finding agrees with a recent study using Bayesian NBR^8^, and is also supported by several studies showing larger tumor size is correlated with an increased risk of a higher number of positive ALNs^10,26^. We may conclude that patients with different tumor sizes might undergo surgery to remove individual axillary nodes for refining treatment. A previous study by Coombs, N suggested a similar perspective^27^. In their results, small tumors less than 20 mm in size tended to potentially benefit from SLNB. In patients with moderate-sized tumors (20–40 mm), the risk of ALNs is relatively high and should be assessed according to tumor characteristics and other work-up results, and patients will probably need a secondary axillary node operation. Patients with a large tumor size (> 40 mm) usually have a high probability of ALN metastases, and complete ALND is a preferred surgical procedure.

Presently, we have entered an era of precision medicine that attempts to be less invasive in our surgical procedures and to lower treatment-related long-term morbidity, with an ultimate goal of improving the quality of life for cancer survivors. The advent of SLNB has drastically decreased the complications of ALND. However, the question of whether SLNB alone is good enough in the setting of metastatic ALNs, or whether ALND is still required to improve cancer-related outcomes, remains to be answered. The perspective that ALND does not improve survival and is therefore not a rational option was derived from the NSABP B-04 trial in which patients received total mastectomy with or without ALND as well as with or without radiation therapy^28^. After a 25-year follow-up, no significant difference was found in the axillary recurrence rate in both treatment groups^29^. This result indicated that there might be a subset of patients who do not need ALND or regional radiation therapy. The ACOSOG Z0010^30^ and NSABP B-32^31^ trials showed that patients with positive ALN by immunohistochemistry staining did not need to proceed to complete ALND without a reduction in overall survival. Since then, evidence has accumulated from studies of the outcomes of patients with involved SLNs who have a low axillary burden do not undergo ALND, and no effects on overall survival and axillary recurrence were found. The IBCSG 23-01 trial demonstrated that omission of ALND did not lead to compromised local recurrence or survival when one or more micro-metastatic SLNs were found in patients with early-stage disease^32^. The ACOSOG Z0011 trial then further showed that in patients with early-stage breast cancer and 1–2 micro- metastatic or macro-metastatic SLNs, treated with breast conservation therapy and subsequently chemotherapy, omission of ALND did not adversely impact locoregional control and survival^33^. The results of the AMAROS trial partly confirmed that mastectomy or conservation surgery in patients with limited positive SLNs after axillary radiotherapy or ALND does not have an effect on axillary recurrence^34^. All of the above clinical trials supported the idea that ALND is unnecessary in these selected patients. Our study showed that about approximately 70% of patients had 0–2 ALN metastases, which may indicate that SLNB was sufficient for these selected patients. However, we cannot forget that the ultimate goal of management is to cure and control cancer. Therefore, preoperatively evaluating patients with a low ALN burden who would not obtain additional benefit from ALND has become very important.

In the present study, the receiver operating characteristic (ROC) analysis in the modeling group and validation group both indicated a good predictive ability. To further justify clinical utility, DCA was applied to assess whether the NBR model would improve our evaluation of 0–2 ALN metastases in validation group. Combination of ROC and DCA analyses, patient with a probability threshold of > 90%, has the greatest chance of avoiding unnecessary ALND.

There are several highlights in the study: (1) To the best of our knowledge, this is the first NBR model for predicting 0–2 ALN metastases in patients with breast cancer. The model is very important for assisting in management axillary surgery in breast cancer patients in the post-Z001 trial era. (2) Our predictive model included seven variables that are routinely available preoperatively, such as primary tumor size, clinical ALN status, histologic grade, ER status, and HER2 status, which can be obtained before surgery. The availability of these data is very important for clinicians to inform patients regarding the possibility of having involved ALNs before undergoing the procedure. (3) Our approach of creating a modeling with data from one hospital and validating it with data from another center is an appropriate measure to reduce bias.

Despite the intriguing findings and strengths in the present study, there are a few limitations that need to be addressed. First, the present study did not include the lympho-vascular invasion variable since it is difficult to identify through a core needle biopsy. Several studies indicated that lympho-vascular invasion was a stronger risk factor associated with ALN metastasis^3,10,35^. The lack of consideration of lympho-vascular invasion in this study may influence the accuracy of the model. Second, the occurrence of node metastasis partly reflects a time-dependent process, as cancer cells are more likely to develop lymphogenic and/or hematogenic metastases with time. The age of a breast tumor is difficult to calculate. However, the tumor size can indirectly reflect tumor age. In this study, tumor size was also included in the final model, which was related to the incidence of ALN metastasis. Therefore, the lack of define tumor age may not affect the outcome. Third, this study has inherent weaknesses because of its retrospective nature. Fourth, this model can only be applied in patients with a single lesion and is restricted to those with unilateral disease.

In conclusion, ALN evaluation preoperatively is an essential element in the management of breast cancer. The shift to less invasive surgical procedures and increased adoption of tailored therapy has been paralleled by the shift from ALND to SLNB and even omitting axillary surgery in selected patients. Our study highlights the distribution of ALN metastasis and the factors that may affect the counts of ALN metastases in breast cancer. Our NBR model suggests the relevance of these important predictors and might help physicians to weigh the risks of involved ALNs and determine appropriate surgical procedures.

## Materials and methods

### Patients

The inclusion criteria of present study were (1) female patients with unilateral breast cancer, (2) patients with pathologically confirmed invasive ductal carcinoma, and (3) patients with complete medical registry and follow-up information. The exclusion criteria were male patients, patients with bilateral or occult breast cancer, patients treated with neoadjuvant chemotherapy, patients with distant metastasis, and patients with pathologically confirmed non-invasive ductal carcinoma. Patients with ductal carcinoma in situ with microinvasion were also excluded due to the difficulty of being diagnosed with core needle biopsy. From Jan 2012 to Dec 2013, 434 consecutive patients with primary breast cancer admitted to The Breast Center in the Cancer Hospital of Shantou University Medical College (SUMC) were reviewed. A total of 212 patients eligible for our study were included in the modeling group (Figure S1A). From Aug 2014 to Dec 2017, 384 consecutive patients with primary breast cancer admitted to the Department of Thyroid and Breast Surgery in the First Affiliated Hospital of SUMC were reviewed. Ultimately, 274 eligible patients were included in the validation group (Figure S1B). The demographic and clinicopathologic characteristics were obtained from the hospital medical records, including age at diagnosis of tumor, primary tumor size based on preoperative physical examination or imaging scans, laterality, tumor location (quadrant), menstrual status, clinical lymph node status, number of ALN metastases, number of ALN dissections, ER status and progesterone receptor (PR) status, Ki67 index, HER2 status, histologic grade and type of surgery.

In the modeling group, ALND was performed when pathologically assessment, through either hematoxylin and eosin staining or immunohistochemistry, revealed SLN involvement. According to the Z0011 trial criterion, ALND was performed only in the positive SLNs postoperatively, excluding those with micro-metastasis^36^.

### Pathology

Pathological examinations of tumor samples were analyzed by two experienced pathologists. The number of ALNs with and without metastases at the final pathology review was recorded. Tumor tissues were obtained from surgical resection specimens. The technique used for histopathological analysis is referred to previous publication^37^. The histological grade scoring for invasive tumors was in line with the Nottingham grading system^38^. Serial section hematoxylin and eosin staining was performed to examine all nodes postoperatively. Immunohistochemistry staining was further performed to determine the presence or absence of micro-metastases (0.2–2 mm cancer foci) when no cancer cells were detected on hematoxylin and eosin staining. In the present study, micro-metastasis was defined as ALN metastasis. ER^39^ and PR^39^ were considered positive if more than 10% of tumor cells demonstrated positive immunostaining. A score of 3 + on immunohistochemistry or amplification on fluorescence in situ hybridization was considered HER2 positivity^40^. According to the Breast Cancer Working Group guidelines, the Ki67 index was determined by the percentage of positive cells among a total number of 1,000 tumor cells, with at least 500 tumor cells being counted^41^. Ki67 was considered positive if ≥ 14% tumor cells had nuclear staining^42^.

### Statistics

The traditional NBR model, commonly known as negative binomial (NB) 2, is based on a Poisson-gamma mixture distribution allowing the modeling of Poisson heterogeneity using a gamma distribution^21^. In NBR, the mean of *u* is calculated by incorporating the exposure time *t* and a set of *k* regressor variables, and the variance function is *μ* + *αμ*^2^. In the present study, the exposure time was set to 1.0. The expression of related quantities is formulated as follows:

1 $${u}_{i}=\mathit{Exp}\left({\beta }_{1}{x}_{1i}+{\beta }_{2}{x}_{2i}+\dots +{\beta }_{k}{x}_{ki}\right)$$

Often, ${x}_{1}\equiv 1$, in which *β*_***1***_ is termed the intercept. The regression coefficients *β*_1_, *β*_2_, …, *β*_k_ are unknown parameters that are estimated from a set of data. Their estimates are symbolized as *b*_1_, *b*_2_, …, *b*_k_. Using this notation, the probability distribution function of the NBR model for an observation *i* is written as follows^43^:

2 $$Pr\left({y}_{i}|{u}_{i}, \alpha \right)=\frac{\Gamma \left({y}_{i}+{\alpha }^{-1}\right)}{\Gamma \left({y}_{i}+1\right)\Gamma \left({\alpha }^{-1}\right)}{\left(\frac{1}{1+\alpha {u}_{i}}\right)}^{{\alpha }^{-1}}{\left(\frac{\alpha {u}_{i}}{1+\alpha {u}_{i}}\right)}^{{y}_{i}}$$

The NB distribution is a two-parameter model with a mean parameter *u* and dispersion parameter α. The function $\Gamma$(·) is the gamma function.

Mann–Whitney U test was used to analyze continuous variables, and the χ^2^ test was used for the comparison of categorical variables. The dependent variable was the counts of metastatic ALNs (discrete numerical variable). The independent variables in the model were age at diagnosis of tumor (continuous variable), and clinical tumor size (continuous variable). Categorical variables included menstrual status, laterality, quadrant, clinical lymph node status, histologic grade, ER/PR status, HER2 status and Ki67 index. The maximum likelihood was used to estimate the regression coefficients^43^.

In the modeling group, we chose the one-way terms options, and set the hierarchical forward to switch with the regression model. We chose a subset size using the maximum likelihood after which the log likelihood was not increased significantly. In the first run, we set the maximum number of variables to find the appropriate number of parameters by looking at changes in the log likelihood. Then, we reset the maximum subset size to this value and made a second run. Next, the variables of primary tumor size, clinical lymph node status, histologic grade, ER status and HER2 status were included in the model. The IRR and 95% CI for each variable were calculated. Then, the model was applied to the validation group by incorporating the eligible variables and calculating each individual patient’s probability of having 0–2 ALN metastases. Finally, ROC analysis was used to evaluate the accuracy and discriminative ability of the models in the training cohort and in the validation cohort. DCA was also performed to estimate the clinical usefulness and benefits^44^. Statistical analyses were performed with the JMP statistical software version 13.2.0 (SAS Institute Inc., Cary, NC, USA), the NCSS 11 statistical software (2016) (NCSS, LLC. Kaysville, Utah, USA, ncss.com/software/ncss), and R software with the rms package (version 3.5.2, http://www.r-project.org). *P* values less than 0.05 from a two-sided test were considered statistically significant.

### Ethical approval and informed consent

This study was reviewed and approved by the Institutional Review Board of the Cancer Hospital of SUMC and the First Affiliated Hospital of SUMC. It was performed in accordance with the ethical standards laid down in the 1964 declaration of Helsinki and all subsequent revisions. All persons mentioned in the paper gave their informed consent prior to inclusion in the study.

## Supplementary Information

Supplementary Table.

Supplementary Figure.

## Data Availability

The datasets used and/or analyzed during the current study are available from the corresponding author on reasonable request.
