# Supplementary material for: A negative binomial regression model for risk estimation of 0–2 axillary lymph node metastases in breast cancer patients
Source: Sci Rep. 2020 Dec 14;10:21856. doi: 10.1038/s41598-020-79016-4 (PMC7736885; doi:10.1038/s41598-020-79016-4)
Supplement: Supplementary file 1 — Supplementary Table. [file 41598_2020_79016_MOESM1_ESM.docx]

**Supplement Table S1** The goodness of fit with negative binomial regression in modeling group

| **Statistic** | **Value** |
| --- | --- |
| Number of patients | 212 |
| Log Likelihood: Max Possible | -268.54 |
| Log Likelihood: Model | -366.51 |
| Log Likelihood: Intercept Only | -440.18 |
| Pseudo *R*-squared | 0.43 |
| Deviance | 195.95 |
| AIC (1) | 745.03 |
| AIC (n) | 3.51 |
| BIC (R) | -907.51 |
| BIC (L) | 765.17 |
| BIC (Q) | 3.56 |
