# Supplementary figures and images for: A negative binomial regression model for risk estimation of 0–2 axillary lymph node metastases in breast cancer patients
Source: Sci Rep. 2020 Dec 14;10:21856. doi: 10.1038/s41598-020-79016-4 (PMC7736885; doi:10.1038/s41598-020-79016-4)

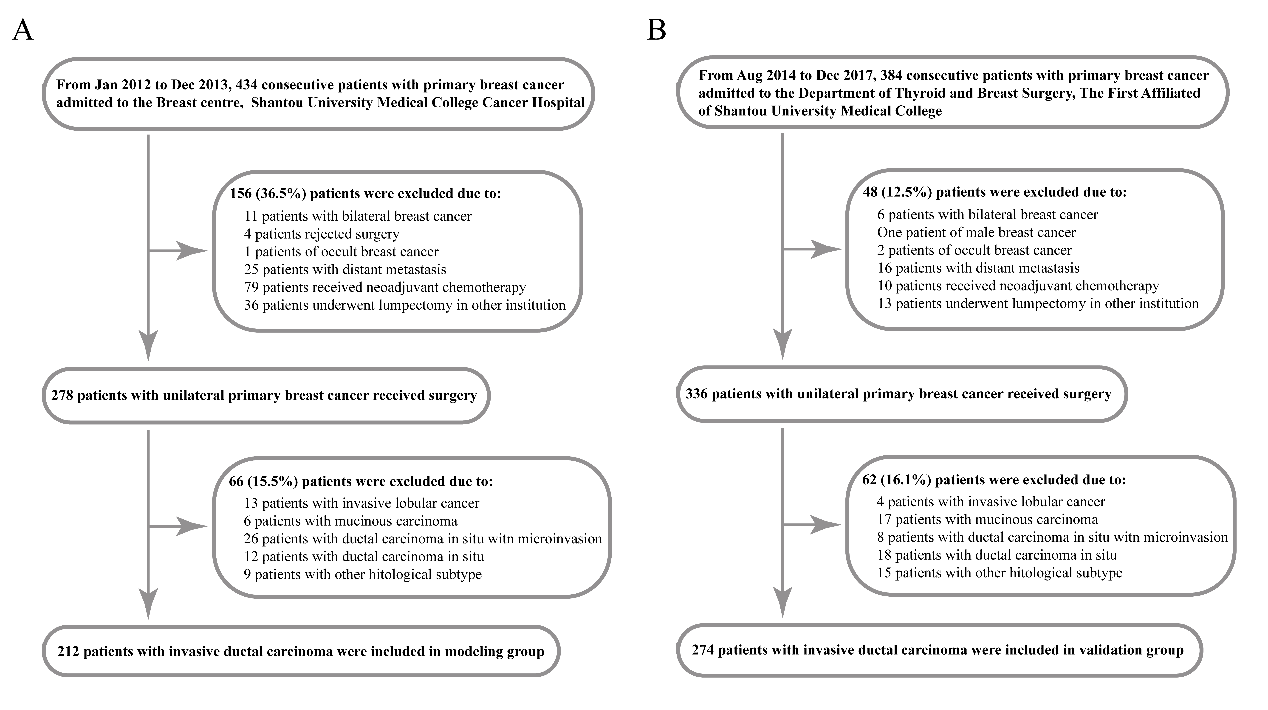


**Supplement Figure S1** Diagram of patients analyzed in this study.

Supplement: Supplementary file 2 — Supplementary Figure. [file 41598_2020_79016_MOESM2_ESM.docx]
